# Temperature variability increases *Trypanosoma cruzi* load but not the extrinsic incubation period in *Triatoma infestans*

**DOI:** 10.1186/s13071-026-07270-y

**Published:** 2026-02-02

**Authors:** Bárbara Álvarez-Duhart, Sabrina Clavijo-Baquet, Lucía Valenzuela-Pérez, Juan Diego Maya, Miguel Saavedra, Sofía Ortiz, Catalina Muñoz-San Martín, Antonella Bacigalupo, Pedro E. Cattan

**Affiliations:** 1 Facultad de Medicina Veterinaria, Universidad San Sebastián, Campus Bellavista, Santiago, Chile; 2 Departamento de Ciencias Biológicas Animales, Facultad de Ciencias Veterinarias y Pecuarias, Universidad de Chile, Santa Rosa 11725, La Pintana, 8820808 Santiago, Chile; 3 Sección Etología, Facultad de Ciencias, Universidad de la República, Montevideo, Uruguay; 4 Departamento de Ecología, Facultad de Ciencias Biológicas, Pontificia Universidad Católica de Chile, Santiago, Chile; 5 Laboratorio de Biología Celular y Molecular, ICBM Facultad de Medicina, Universidad de Chile, Santiago, Chile; 6 ICBM Instituto de Ciencias Biomédicas Facultad de Medicina, Universidad de Chile, Santiago, Chile; 7 School of Biodiversity, One Health and Veterinary Medicine, University of Glasgow, Glasgow, Scotland, UK

**Keywords:** Climate change, Infectious, Chagas disease, Vector-borne diseases

## Abstract

**Background:**

*Trypanosoma cruzi*, the etiologic agent of Chagas disease, is transmitted via the dejections of triatomine insects such as *Triatoma infestans.* Parasite development inside the vector depends on temperature, which determines the extrinsic incubation period (EIP) and modulates the parasite load. As global warming is expected to increase mean temperatures and thermal variability, these shifts may influence vector competence.

**Methods:**

*Triatoma infestans* individuals were experimentally infected with *T. cruzi* Dm28c strain and then exposed to four thermal regimes: two constant (18 °C and 27 °C) and two fluctuating (18 ± 5 °C and 27 ± 5 °C). Parasite load in the dejection samples was quantified by quantitative PCR over 42 days and the time to the first positive dejection determined to estimate the EIP.

**Results:**

Higher temperatures significantly shortened the EIP, with mean values of 18.6 days at 18 ± 0 °C, 17.3 days at 18 ± 5 °C, 9.6 days at 27 ± 0 °C and 11.0 days at 27 ± 5 °C. Temperature variability did not affect the EIP but it did increase parasite load under cold conditions. Parasite load showed a bell-shaped curve, peaking earlier and at higher levels at warmer temperatures. A larger volume of ingested blood also reduced the EIP, especially under cold treatments.

**Conclusions:**

Rising temperatures accelerate *T. cruzi* development within *T. infestans*, potentially enhancing vector competence under climate change scenarios. Although temperature variability did not affect the EIP, it increased parasite load, particularly under cold conditions, which is a relevant result considering that low temperatures have historically limited the vector and Chagas disease transmission. Temperature variability—not only mean warming—can modulate parasite development. Our results therefore provide novel and relevant insights into how climate change may alter vector-borne disease dynamics.

**Graphical Abstract:**

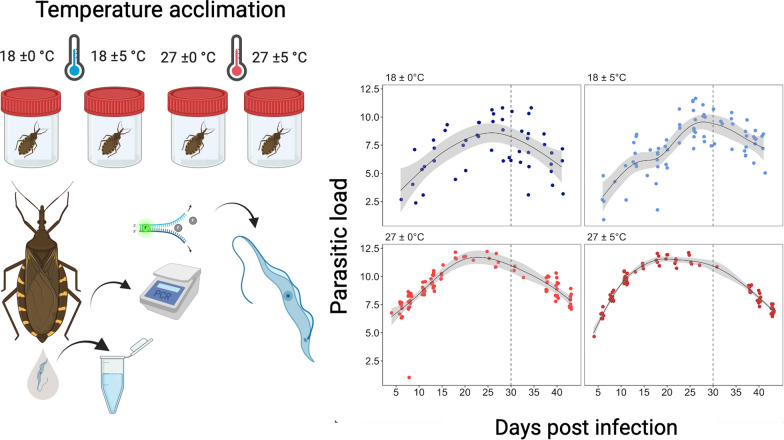

**Supplementary Information:**

The online version contains supplementary material available at 10.1186/s13071-026-07270-y.

## Background

The relationship between climate change and infectious diseases is a pressing ecological and global health concern [1, 2]. Vector-borne diseases are especially susceptible to temperature because their transmission rate and incidence are related to the biological traits of vectors, which are ectothermic arthropods [2]. Climate change is expected to affect multiple vector-borne diseases, including dengue fever [3], chikungunya virus [4], leishmaniasis [5], malaria [6] and Chagas disease [1]. The influence of temperature on key vector traits, such as biting rate, reproduction, development, survival, and the probability of becoming infectious after biting an infectious host (i.e. vector competence) has been reported for several disease vectors [2, 7–9]. In brief, pathogen transmission is promoted within optimal thermal ranges and constrained beyond the lower and upper thermal activity limits [2, 10].

Climate change projections indicate long-term increases in mean environmental temperature and greater temperature variability across daily, monthly and seasonal scales [11]. Thermal variability affects ectotherm survival, reproduction and performance in nonlinear ways, often enhancing performance below the thermal optimum while reducing it above this threshold [12–14]. Despite growing evidence that temperature variability can modulate ectotherm performance, its consequences for vector competence and pathogen development remain poorly understood, hindering predictions of how global warming will alter the distribution and incidence of vector-borne diseases [2, 3, 15].

The extrinsic incubation period (EIP) is the time required for a pathogen or parasite to develop from its entry into the vector until the first forms capable of infecting another host appear [16]. The EIP influences the incidence of vector-borne diseases [17–19] because a shorter EIP can increase pathogen transmission when key parameters are not limiting [8]. Together with other temperature-sensitive traits, such as vector survival and biting rate, EIP determines the basic reproductive number ®_0_) for vector-borne diseases [20] and is also included in the vectorial capacity equation [18, 19, 21]. Therefore, even small changes in EIP may have a significant impact on the results of mechanistic models based on these equations [22–24], as has been demonstrated for dengue [19], malaria [25] and bluetongue [26]. In general, the EIP has been shown to respond not only to mean temperature changes but also to temperature variability in several vector–pathogen systems [17, 27, 28]. These responses to temperature highlight the role of thermal conditions in shaping vector ecology and behavior, with direct implications for parasite development and transmission risk.

Chagas disease is a neglected tropical disease caused by the flagellate protozoan *Trypanosoma cruzi*, with 6 million people estimated to be infected worldwide, predominantly in endemic areas of Latin America [29, 30]. The primary mode of transmission for this protozoan involves the contact of mammalian mucous membranes or wounds with contaminated feces or urine from hematophagous insect vectors belonging to the subfamily Triatominae (Hemiptera: Reduviidae) [31, 32]. Transmission can also occur through other routes, such as blood transfusions, congenital transmission, oral route, organ transplants and during laboratory accidents [33–36]. *Triatoma infestans* is the most important Chagas disease vector in southern South America [37]. Wild foci of *T. infestans* have been documented in Bolivia [38], Argentina [39], Paraguay [40] and Chile [41]. In some countries, the presence of wild foci may hinder vector elimination efforts because insects from these foci can recolonize peridomestic and domestic habitats [41–43]. Climate warming could further exacerbate this process by increasing vector population sizes and activity levels, potentially generating more flight-dispersing individuals capable of invading houses from both sylvatic and domiciliary sources [44, 45]. In addition, winter temperature has been proposed as a limiting factor in terms of population growth [46–48]. Higher temperatures increase the feeding behavior of *T. infestans*, with temperature variability showing nonlinear effects on feeding frequency [47]. Temperature is also positively associated with *T. infestans* flight initiation [48].

*Triatoma infestans* inhabits peridomestic habitats that can buffer temperature fluctuations. Its thermal preference lies between 25 °C and 29 °C [49], which is easily reached inside houses, but shows activity between 18 °C and 42 °C [50]. Currently, the minimum mean winter temperature in the central region of Chile, where wild *T. infestans* foci have been reported, is 10.7 °C, and the minimum mean temperature in the coldest month is 4 °C, below the *T. infestans* activity range [51]. However, climate change projections estimate a temperature increase ranging from 2 to 5 °C in the worst-case scenario [51]. Considering the buffering effect of peridomestic structures and houses, which can reduce thermal extremes by up to ± 5−8 °C [52], minimum winter temperatures could fall within the activity range of *T. infestans,* potentially expanding habitat suitability beyond its historical distribution*.* Nevertheless, the distribution and presence of *T. infestans* results from a complex interplay between ecological, climatic and behavioral factors [53].

Temperature is a major abiotic factor in *T. cruzi*–triatomine interactions [54, 55]. The development of *T. cruzi* and its relationship with temperature have been studied under field and laboratory conditions, revealing a positive relationship between environmental temperature and the concentration of infectious forms of *Triatoma proctata* [56], *T. infestans* [57] and *Rhodnius prolixus* [58]. Increased temperature reduces parasite incubation within the vector [57]. However, despite the importance of temperature variability in climate change, its effect on parasite development and EIP in triatomines has not been tested under controlled conditions. Therefore, in this study, we aimed to evaluate the influence of temperature and its variability on the EIP and parasite load of *T. cruzi* in *T. infestans* and the probability that insects (infected or not) produced *T. cruzi*-positive dejections over time.

## Methods

A total of 144 fourth-instar *T. infestans* nymphs were obtained from a colony that had been maintained for 2 years at the Laboratorio de Ecología, Facultad de Ciencias Veterinarias y Pecuarias, Universidad de Chile. This colony was originally established in 1950 at the Faculty of Medicine from the same university. The fourth instar is easily manageable and can transmit *T. cruzi* to vertebrate hosts [59]. The specimens were maintained individually in plastic flasks (3.8 × 6.8 cm) inside climatic chambers maintained at 27 °C and 50–70% humidity under a photoperiod of 12:12 (light:dark) [60]. The triatomines were fasted for 15 days prior to feeding on infected mice (*Mus musculus*).

### Triatomine infection with *T. cruzi*

Triatomines were infected by feeding on mice previously inoculated with *T. cruzi* clone Dm28c (DTU-TcI) under controlled laboratory conditions. The trypomastigote forms of *T. cruzi* were cultivated in Vero cells (60–70% confluence), 5 ml of RPMI 1640 culture medium (Biological Industries, Beit Haemek, Israel) with 10% fetal bovine serum, 2 ml of trypsin, 1 ml of penicillin and 100 μg/ml of streptomycin (Biological Industries), as previously described [61]. Fourteen 8-week-old female BALB/c mice were inoculated once by intraperitoneal injection with 1000 parasites suspended in 1 ml of RPMI 1640 medium, and the parasite concentration was determined by light microscopy using a Neubauer chamber. After inoculation, mice were housed in cages at 20–25 °C and 40–70% relative humidity, with free access to water and food, following the approved bioethics protocol (no. 19262-MED-UCH). Mouse parasitemia was evaluated by measuring parasite concentration and viability in 0.1 ml of blood extracted from the tail vein, by light microscopy (40×) with a Neubauer chamber every 3 days from day 7 post-infection until 80,000–500,000 trypomastigotes/ml was reached, ensuring triatomine infection. Once this infection level was reached, the insects were fed on the *T. cruzi*-inoculated mice. Mice were sedated following the bioethics protocol (no. 19262-MED-UCH) with a dose of ketamine 100 mg/ml (mouse dose 100 mg/kg) in association with xylazine 5 mg/ml (mouse dose 10 mg/kg) and then placed inside a plastic box (32 × 21 × 14 cm) over a heating pad. Triatomines were fed during the scotophase by placing them in the same plastic box as the mouse for 30 min in the dark. Twelve insects, after each was individually marked with colored paint [62], were placed with a mouse for feeding. Each triatomine was weighed before and immediately after feeding using a BOECO model BAS-31 analytical balance with a precision of 0.0001 g (Boeckel & Co. GmbH & Co. KG, Hamburg, Germany) to check for blood ingestion during the experimental procedure. All insects were included regardless of the blood volume ingested.

### Temperature treatments

Temperature treatments were chosen based on the average temperature of the central zone of Chile [51], where populations of the species have historically been detected, both in domestic and wild environments [63, 64], and the temperature of the insect microsite habitat [46]. The lowest acclimation temperature considered was the mean winter temperature (10.7 °C), plus a 5 °C microhabitat buffer and an additional 2 °C that reflected projected temperature increases under climate change, with and without the inclusion of temperature variability (i.e. 18 °C ± 5 °C and 18 °C ± 0 °C, respectively). We also acclimated individuals to their optimal temperature, with and without temperature variability (i.e. 27 °C ± 5 °C and 27 °C ± 0 °C, respectively), which is the inside-house temperature [50]. To maintain the desired photoperiod, lights were switched on at 7:00 h and turned off at 19:00 h. In the variable temperature treatments, the rise in temperature occurred during the light hours, with the temperature starting to increase linearly at 7:00 h, reaching a maximum at 8:00 h (32 °C and 23 °C for the warm and cold treatments, respectively), then remaining constant until 19:00 when it started to decrease, reaching a minimum at 20:00 h (22 °C and 13 °C for the warm and cold treatments, respectively). Additionally, the temperature range used in these experiments was well within the thermotolerance range of the species, thus avoiding individual mortality [13]. The diurnal variation range selected was conservative with respect to the diurnal daily variation of Chile’s Central Region [65], but was chosen because the animals inside the climatic chambers cannot use behavioral thermoregulation. These temperatures have been previously studied, so they will allow the integration of information for future studies on the effects of climate change on *T. infestans* [12, 47]. Environmental chambers were set to the target temperatures with an accuracy of ± 1 °C and a precision of 0.2 °C (PiTec Puig Ingeniería y Tecnología; Av. Campos de Deportes 851, Ñuñoa, Región Metropolitana; Postcode 7750459), under photoperiod and humidity conditions identical to those of the maintenance conditions. Temperature and humidity were monitored daily using climatic chamber sensors.

After feeding on infected mice, all triatomines were randomly allocated to one of the four thermal treatments (i.e. 18 ± 0 °C, 18 ± 5 °C, 27 ± 0 °C and 27 ± 5 °C). Each treatment group comprised 36 individuals, with each insect housed separately in a plastic vial (3.8 × 6.8 cm). Each vial was labeled with a unique identification code indicating the individual insect, its assigned thermal treatment and source mouse. The individual identification system was used throughout the experiment. The total maintenance time under these treatments was 42 days post-infection (p.i.).

### Dejection sampling

Every 2 days, each individual was transferred to a new flask labeled with its corresponding identification code and date. Simultaneously, the nymphal state of all the individuals in the experiment was recorded. Excretion samples were placed in Eppendorf tubes, labeled and recorded according to the identification code, date of collection and number of fecal samples collected in the flask. Each fecal sample was diluted with nuclease-free water (40 µl per excretion), and the contents of the solution were subsequently transferred to 1.5-ml Eppendorf tubes labeled with the insect ID and collection date. These tubes were then stored at − 20 °C until DNA extraction. Kollien and Shaub [66] observed that a short-term starvation period of 30 days led to the initial appearance of deceased flagellates in the rectum of triatomines, whereas a starvation period of 90 days resulted in a mortality rate of 99.5% of *T. cruzi* in the rectum. Thus, triatomines were fed on non-infected laboratory mice at day 30 p.i. using the same feeding protocol described for triatomine infection. We measured the weight before and after feeding and recorded the nymphal state. After the experimental period (42 days), the insects were euthanized by freezing for a minimum of 48 h following bioethical protocol no. 19262-MED-UCH.

### *Trypanosoma cruzi* detection in dejections

*Trypanosoma cruzi* DNA was extracted from *T. infestans* dejections using the innuPREP Blood DNA Mini Kit (Analityk Jena GmbH+Co. KG, Jena, Germany), following the manufacturer’s instructions. All samples were co-extracted with 1 pg/μl of a 183-bp synthetic sequence from *Arabidopsis thaliana* (Brassicales: Brassicaceae), synthesized using gBlocks Gene Fragments (Integrated DNA Technologies, Inc. [IDT], Coralville, IA, USA) and used as an exogenous internal amplification control to evaluate the carryover of inhibitors and DNA loss in the extraction process.

The quantitative PCR (qPCR) assays amplified the satellite nuclear conserved region of *T. cruzi* with the primers Cruzi 1 (5′–ASTCGGCTGATCGTTTTCGA–3′) and Cruzi 2 (5′–AATTCCTCCAAGCAGCGGATA–3′) [68] in a final reaction volume of 20 μl, containing 5 μl of template DNA, 5× HOT FIREPol EvaGreen qPCR Mix Plus (Solis BioDyne, Tartu, Estonia), 0.3 μM of each primer and nuclease-free water. The qPCR assays were carried out in a Rotor-Gene Q thermal cycler (QIAGEN, Hilden, Germany); the cycling conditions consisted of a pre-incubation for 15 min at 95 °C, followed by 40 cycles of a denaturation step at 95 °C for 15 s, a hybridization step at 60 °C for 20 s and a final extension step at 72 °C for 20 s. The emitted fluorescence was recorded at the end of each cycle, and a melting curve was generated at the end of the program, with a ramp from 72 °C to 95 °C, increasing by 1 °C in each step, waiting for 90 s of pre-melting conditioning in the first step, and 5 s for each subsequent step. Each reaction was carried out with a negative control (DNA from infection-free triatomine dejection), a positive control (*T. cruzi* DNA quantification standard) and a no-template control (nuclease-free water). All samples were analyzed in duplicate.

The DNA standard curve for absolute quantification was obtained from the genomic DNA of *T. cruzi* strain DM28c. The calculation was performed considering that a parasite cell contains approximately 200 fg of DNA [67, 68]. Serial 1:10 dilutions were made with nuclease-free water to cover a range of 10^6^ to 0.1 parasite equivalents/ml. All samples were co-extracted with 1 pg/μl of a 183-bp sequence from the tonoplast intrinsic protein 5;1 (TIP 5;1) of *A. thaliana* to normalize the parasite load, as previously described [69]. Quantification of the parasite equivalents from the DNA samples was performed by considering the amplification of the *T. cruzi* DNA standard curve, and the results were normalized according to the results of the exogenous internal amplification control (IAC).

### Statistical analysis

#### Extrinsic incubation period

Only the first positive dejection sample for each individual was used to estimate the effect of temperature on the EIP of *T. cruzi* in *T. infestans* (total *n* = 108; 18 ± 0 °C, *n* = 23; 18 ± 5 °C, *n* = 28; 27 ± 0 °C, *n* = 29; 27 ± 5 °C, *n* = 28). Generalized linear models (GLM) with a gamma function were fitted with EIP as a response variable and temperature treatment (T), individual body mass before infection (mb), ingested blood during infection (bi; estimated by subtracting the weight before and after feeding) and mouse parasitemia (Pm) as predictors (Additional file 1: Table S1). Although a correlation between body mass and blood ingested has been reported in previous studies [47], no such relationship was detected in our data (Pearson’s *r* = − 0.105). Model adequacy was evaluated using the likelihood ratio test (LRT) [70], and model selection was based on the Akaike information criterion (AIC) [71]. Post-hoc comparisons among temperature treatments were performed using Tukey’s test with Shaffer-adjusted *p*-values.

#### Parasite load in* T. infestans* dejections

All positive dejection samples were included in this analysis (*n* = 307; 18 ± 0 °C = 50, 18 ± 5 °C = 74, 27 ± 0 °C = 97 and 27 ± 5 °C = 86). Parasite load (Par-eq_ml_) was analyzed using generalized additive models (GAM) with a log-normal distribution. Similar to the GLM, predictors included were temperature treatment (T), individual body mass before infection (mb), ingested blood during infection (bi), time in days of the collected sample post-infection (Days) and mouse parasitemia (Pm). Nonlinear effects of T, bi and mb on parasite load were modeled using cubic splines (for details see Additional file 1: Table S1). We also included a random effect for triatomine individuals (ID) to account for the pseudo-replication of data. The model was selected according to the AIC [71].

#### Probability of positive dejections

All qPCR results were analyzed from dejection samples (*n* = 438) as binary response variable (positive or negative for *T. cruzi)*. All insects that fed on infected blood were included, regardless of whether they produced positive or negative dejections. The probability of a positive dejection over time under different temperature treatments was analyzed using the GAM for location, scale, and shape (GAMLSS) [56, 72–77]. The response variable was the outcome of qPCR (positive or negative), while predictors included were temperature treatment (T), individual body mass before infection (mb), ingested blood during infection (bi), mouse parasitemia (Pm), a cubic spline of the time (Days) and their interactions (Additional file 1: Table S1). We also included a random effect for triatomine individuals (ID) to correct the pseudo-replication of the data given by the effect of repeated measurements. Modeling with GAMLSS provides additional flexibility for a binary response variable [73] (for more details, see Additional file 1: Table S1. All analyses were performed in R version 2.13.0 [74] using RSTUDIO and the ggplot2, lmtest, multcomp, mgcv, pROC and gamlss packages.

## Results

A total of 144 individual insects were included in the study, of which 108 (75%) were positive and 36 (25%) were negative. A total of 499 samples were collected, including 326 positive and 173 negative samples. At 18 ± 0 °C, 28 of 36 insects produced 78 samples; at 18 ± 5 °C, 33 of 36 insects produced 117 samples; at 27 ± 0 °C, 31 of 36 individuals produced 117 samples; and at 27 ± 5 °C, all 36 individuals produced 126 samples.

### Extrinsic incubation period

**Fig. 1:**
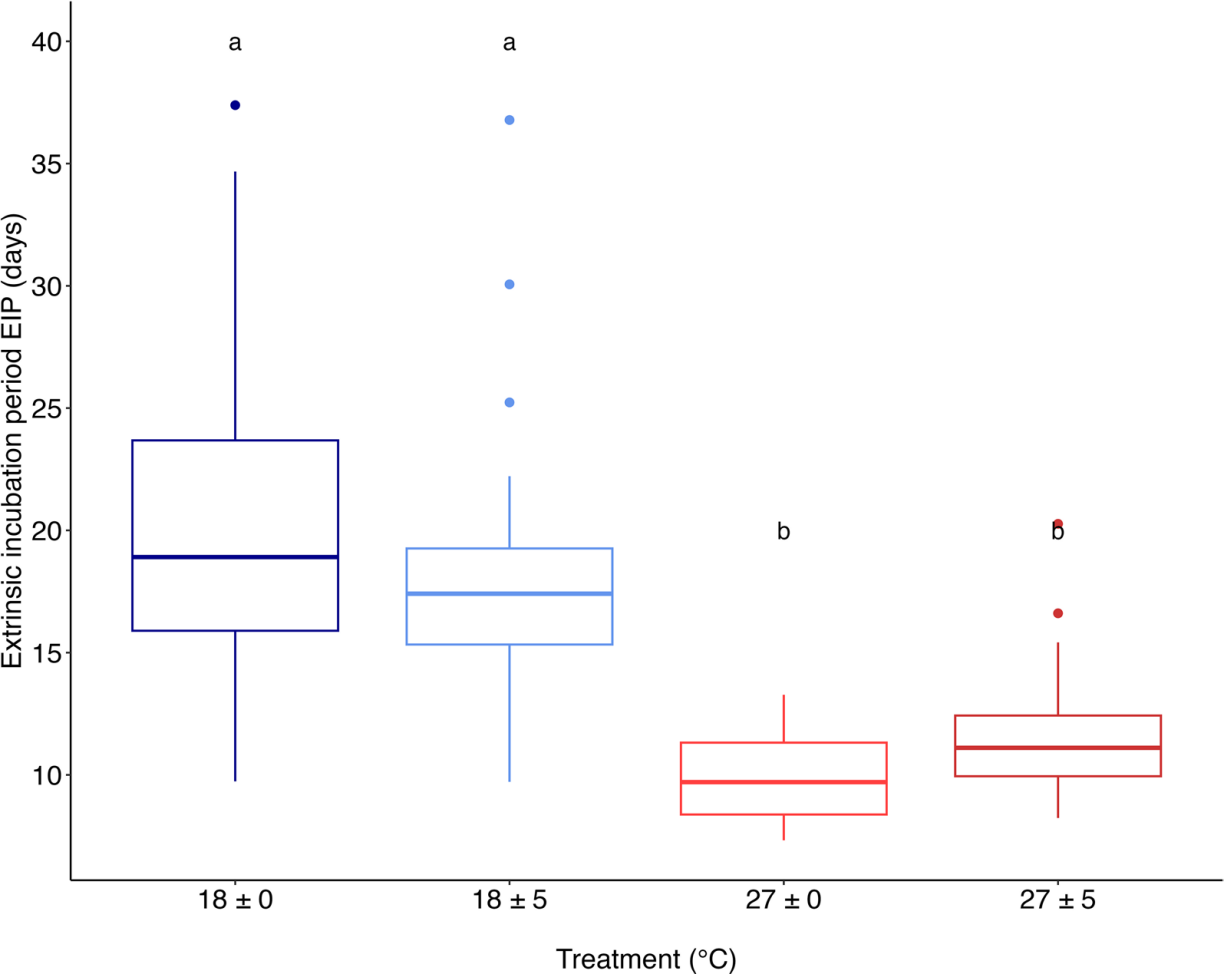
Boxplot of extrinsic incubation period (EIP, measured as days post-infection) of *Trypanosoma cruzi* in *Triatoma infestans* exposed to four temperature treatments 18 ± 0 ºC (*n* = 23); 18 ± 5 ºC (*n* = 28); 27 ± 0 ºC (*n* = 29); 27 ± 5 ºC (*n* = 28). The lowercase letters above each bar show the results of the a posteriori test (Tukey’s test). The boxplot shows no significant differences between the groups at 18 ± 0 ºC and 18 ± 5 ºC, indicated by the same letter ‘a’ above the respective bars, nor between the groups at 27 ± 0 ºC and 27 ± 5 ºC, indicated by the same letter ‘b’ above the respective bars

The best-fitting model indicated that thermal treatment had a significant effect on the EIP (Fig. 1; Additional file 1: Table S2). Relative to the reference group (18 ± 0 ºC), both the warm constant (27 ± 0 ºC) and warm variable (27 ± 5 °C) treatments were associated with shorter EIP values (as is shown in Fig. 1). The GLM showed a good fit to the data (AIC 698.34; Table 1), indicating that the selected predictors significantly contributed to explaining the variation in the extrinsic incubation period of *T. cruzi* in *T. infestans*.

**Table 1:** Summary of the best-fitting generalized linear model for *Trypanosoma cruzi* extrinsic incubation period in *Triatoma infestans*

Predictors	Estimate	Standard error	*t* value	*p*-value	Size effect (days)^a^
Intercept (18 ± 0 ºC)^b^	− 0.007	0.012	− 0.534	0.594	18.59
18 ± 5 ºC^b^	0.004	0.007	0.632	0.529	17.30
27 ± 0 ºC^b^	0.050	0.011	4.525	< 0.05	9.63
27 ± 5 ºC^b^	0.037	0.010	3.819	< 0.05	11.01
bi^c^	0.786	0.189	4.157	< 0.05	0.98
mb^c^	0.975	0.294	3.320	< 0.05	0.22
bi × mb^c^	− 12.918	4.044	− 3.195	< 0.05	-

^a^The size effect was calculated as 1/(intercept estimate + estimate of the category). The volume of blood ingested decreased the extrinsic incubation period in all treatments

^b^Thermal regimes. The groups were defined as: 18 ± 0 °C, cold constant treatment; 18 ± 5ºC: cold variable treatment; 27 ± 0 °C, warm constant treatment; 27 ± 5ºC: warm variable treatment

^c^Predictors included in the generalized linear model. bi, ingested blood; mb, body mass before infection

**Fig. 2:**
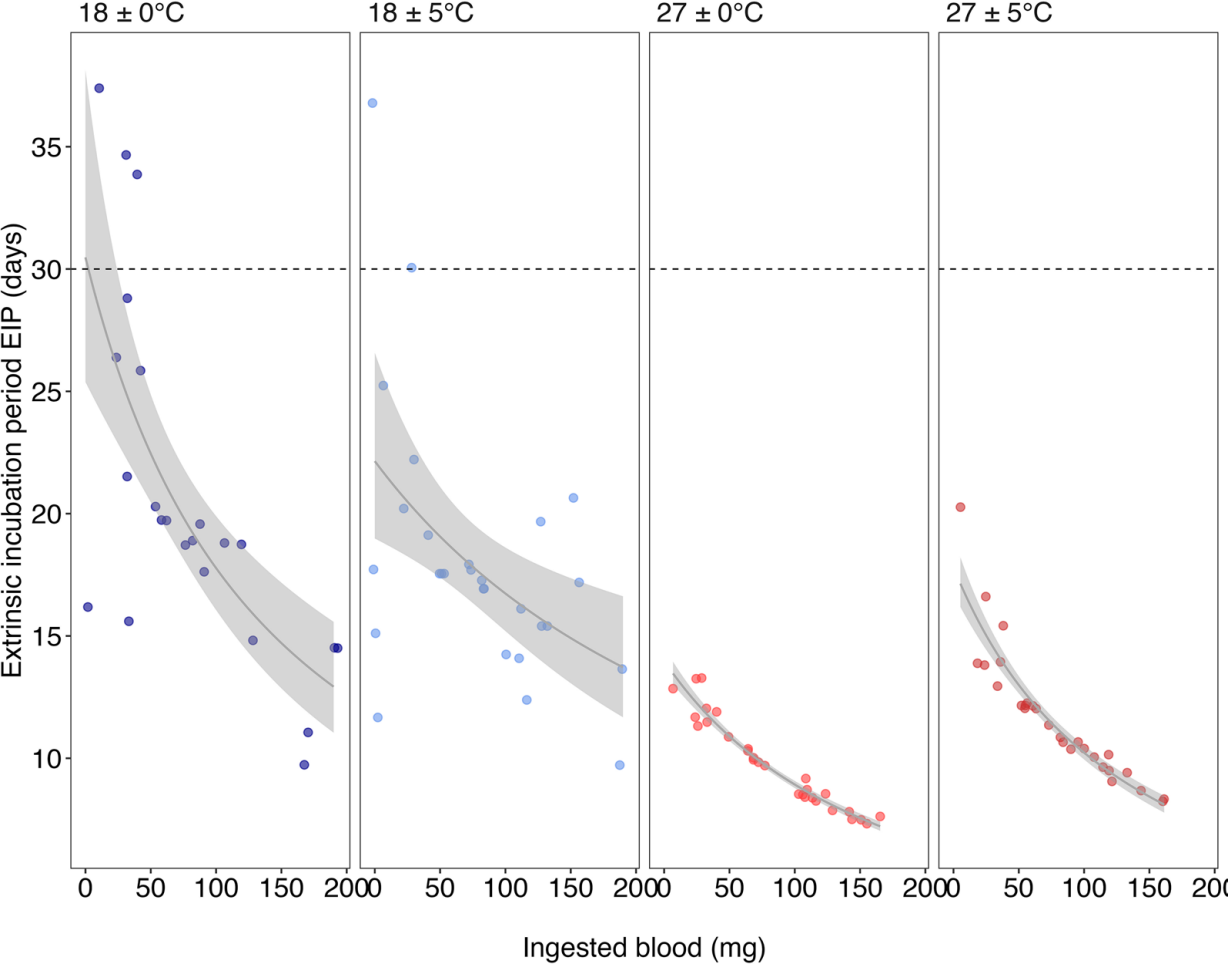
Predicted extrinsic incubation period (EIP, in days) of *Trypanosoma cruzi* in *Triatoma infestans* as a function of ingested infective blood volume (bi, in mg), modeled using a generalized linear model. Each point represents the first positive detection of an individual. Treatments are shown in different panels: constant cold (18 ± 0 ºC, blue, *n* = 23), variable cold (18 ± 5 ºC, light blue, *n* = 28), constant warm (27 ± 0 ºC, red, *n* = 29) and variable warm (27 ± 5 ºC, dark red, *n* = 28). The shaded area indicates the standard error of the mean of the predicted EIP. The dotted horizontal lines indicate the refeeding day

Tukey’s post-hoc tests revealed significant differences between the 27 ºC and 18 ºC treatments, either the constant or variable treatments (Additional file 1: Table S3; Fig. 1). However, no significant differences were detected between constant and thermal variable treatments within the same mean temperature (Fig. 1; Additional file 1: Table S3). The predicted values for the EIP were 18.59 days (± 3.27) for the cold constant treatment, 17.30 days (± 2.86) for the cold variable treatment, 9.63 days (± 1.28) for the warm constant treatment and 11.01 days (± 1.49) for the warm variable treatment (Table 1). An increase in ingested blood infected with *T. cruzi* (bi) reduced the EIP, and the effect was greater for cold treatments (Fig. 2; Table 1).

### Parasite load in dejection samples of *T. infestans*

**Fig. 3:**
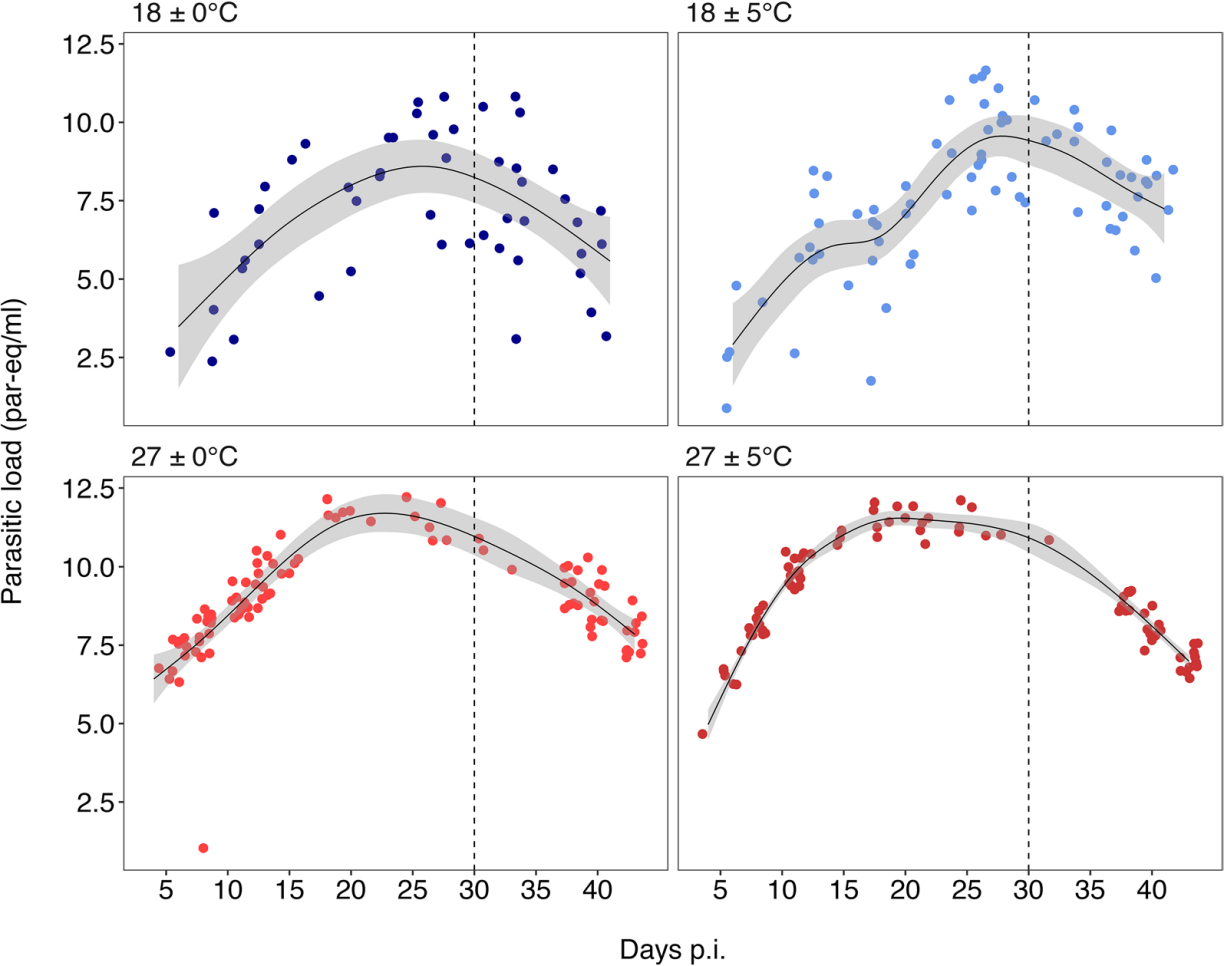
*Trypanosoma cruzi* parasite load (in *Triatoma infestans* excreta samples over time (Days) by temperature treatment from the best-fitting model (*n* = 307). Cold constant temperature regime is shown in blue (18 ± 0 ºC, *n* = 50); cold variable temperature, in light blue (18 ± 5 ºC, *n* = 74); warm constant temperature, in red (27 ± 0 ºC, *n* = 97); warm variable temperature in dark red (27 ± 5 ºC, *n* = 86). Dotted vertical lines indicate the time of refeeding with an uninfected blood meal (day 30). The shaded area shows the standard error of the mean estimated from the best-fit model. Note that parasite load units are shown in parasites equivalent (par-eq) to 200 fg of DNA. p.i., Post-infection

The parasite load varied with temperature treatments (T), time p.i. (Days), body mass (mb) and the interaction term between mb and ingested blood at the time of infection (bi) (Table 2). The selected smooth term (Days, bs = "cs," by = T) indicates that parasite load changed over time differently among T, and this effect was significant for all four treatments (Table 2). Thus, parasite load followed a bell-shaped temporal pattern: it started low, peaked between 20 and 30 days and then declined (Fig. 3). Compared to the 18 ± 0 °C treatment, insects maintained at 27 ± 0 °C and 27 ± 5 °C exhibited significantly higher parasite loads, whereas no significant difference was observed for the 18 ± 5 °C treatment (Table 2; Fig. 3). The interaction between body mass and the amount of blood ingested was significant in all treatments except for 27 ± 5 °C (Table 2) and described different bell-shaped relationships across treatments (Additional file 1: Figure S1). This interaction was strongest in individuals with low body mass, where insects ingesting intermediate volumes of blood exhibited higher parasite loads than those ingesting smaller volumes of blood. This effect weakened with increasing body mass and was reversed in the heaviest individuals (Additional file 1: Figure S1). Altogether, the best-fitting model indicated that parasite load was shaped by time after infection (Day), ingested blood (bi), temperature treatment (T) and body mass (mb) (Table 2; Additional file 1: Table S4). The GAM explained a substantial proportion of the variance (deviance explained = 64.3%; adjusted *R*^2^ = 0.583; AIC = 1448.64;* n* = 307).

**Table 2:** Generalized additive model estimates and significance of the variables in the best-fitting model for the parasite load of *Trypanosoma cruzi* in *Triatoma infestans*

Parametric coefficients:				
Variable^a^	Estimate	Standard error	*t*-value	*p*-value
Intercept (18 ± 0 ºC)	5.210	0.582	8.956	< 0.05
18 ± 5 ºC	0.031	0.704	0.044	0.9647
27 ± 0 ºC	2.575	0.770	3.343	< 0.05
27 ± 5 ºC	2.527	0.751	3.365	< 0.05

Approximate significance of smooth terms:				
Smooth terms^a^	Effective* df*	Reference* df*	*F*	*p*-value
s(ID)	0.977	1.000	7.173	< 0.05
s(Day): 18 ± 0 ºC	3.002	9.000	2.986	< 0.05
s(Day): 18 ± 5 ºC	6.489	9.000	6.806	< 0.05
s(Day): 27 ± 0 ºC	2.952	9.000	5.969	< 0.05
s(Day): 27 ± 5 ºC	4.049	9	8.036	< 0.05
te(mb,bi): 18 ± 0 ºC	8.157	19	2.755	< 0.05
te(mb,bi): 18 ± 5 ºC	8.711	20	2.082	< 0.05
te(mb,bi): 27 ± 0 ºC	4.575	21	1.041	< 0.05
te(mb,bi): 27 ± 5 ºC	1.945	20	0.138	0.230
*R*-sq.(adj) =	0.583	Deviance explained =	64.3%	*n* = 307

Generalized additive model variables: Estimate, Estimate of the selected model; standard error; *t*-value; *p*-value; Effective* df*; Reference* df*;* F*, F-tests on smooth terms; *p*-value, *p*-value of smooth terms

^a^18 ± 0 °C, cold constant treatment; 18 ± 5 °C, cold variable treatment; 27 ± 0 °C, warm constant treatment; 27 ± 5 °C, warm variable treatment; s, spline; ID, triatomine individuals; Day, time measured in days; mb, body mass before infection; bi, ingested blood;* R*-sq.(adj), adjusted* R*-squared value

### Probability of positive dejections

**Fig. 4:**
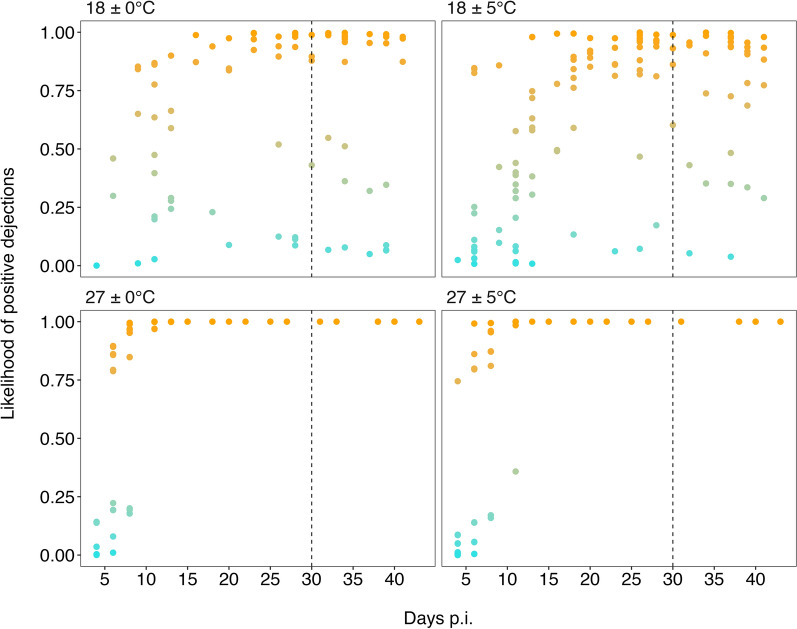
Probability of positive dejections of *Trypanosoma cruzi* in *Triatoma infestans* by temperature treatment. Probability is represented by a gradient palette from turquoise, representing the minimum probability of positivity, to orange, representing the maximum probability of positivity. Dotted vertical lines indicate the time of refeeding with an uninfected blood meal (day 30). All insects that fed on infected blood were included, regardless of whether the dejection samples tested positive, and each point represents a dejection sample obtained from 36 insects per treatment. Sample size per treatment: 18 ± 0 ºC, *n* = 78; 18 ± 5 ºC, *n* = 117; 27 ± 0 ºC, *n* = 117; 27 ± 5 ºC, *n* = 126. p.i., Post-infection

Significant differences were found in the probability of obtaining *T. cruzi*–positive dejections between the warm and cold temperature treatments. However, no significant differences were detected between constant and variable thermal treatments within the same mean temperature (27 ± 0 °C and 27 ± 5 °C; 18 ± 0 °C and 18 ± 5ºC) (Table 3; Fig. 4).

**Table 3:** Parameters of the generalized additive model for location, scale and shape (GAMLSS) selected model

Coefficients^a^	Estimate	Standard error	*t*-value	*p*-value
Intercept (18 ± 0 ºC)	− 3.136	0.477	− 4.038	< 0.05
18 ± 5 ºC	0.285	0.667	0.293	0.769
27 ± 0 ºC	− 5.474	2.573	− 2.317	< 0.05
27 ± 5 ºC	− 8.275	2.187	− 3.984	< 0.05
cs(Days)	0.151	0.020	4.682	< 0.05
18 ± 5 ºC:cs (Day)	− 0.001	0.029	− 0.040	0.967
27 ± 0 ºC:cs (Day)	1.317	0.335	3.894	< 0.05
27 ± 5 ºC:cs (Day)	1.612	0.272	5.756	< 0.05

Parameters of the generalized additive model for location, scale and shape (GAMLSS) selected model: Estimate of the selected model; standard error;* t*-value;* p*-value

^a^ 27 ± 0 °C, warm constant treatment; 18 ± 0 °C, cold constant treatment; 18 ± 5 °C, cold variable treatment; 27 ± 5 °C, warm variable treatment; cs, cubic spline; Day, time in days

The best-fitting model for the probability of *T. cruzi*-positive dejections included a cubic spline for time (Days) interacting with temperature treatments (T) (Additional file 1: Table S5), showing differences between warm and cold treatments (Fig. 4; Table 3). The probability of positive dejections was not different between treatments with the same mean temperature (Table 3; Fig. 4).

At warm temperatures, the probability of positive samples approached 1 and stabilized at approximately 10 days p.i. (Fig. 4). In contrast, insects maintained under cold temperatures exhibited lower and more variable probabilities throughout the experimental period (Fig. 4).

During the refeeding procedure with uninfected mice (dotted vertical black line on Fig. 4), the mean blood ingested by individuals was 0.037 g (± 0.09) at 18 ± 5 °C, 0.046 g (± 0.05) at 18 ± 0 °C, 0.040 g (± 0.04) at 27 ± 0 °C and 0.068 g (± 0.07) at 27 ± 5 °C. Feeding was considered when > 0.0001 g was consumed. Some individuals died during the treatment and were removed from the experiment; therefore, not all treatments included 36 individuals. Nymphal molts occurred primarily in the warm treatments at 27 ± 0 °C (13 individuals molted) and 27 ± 5 °C (12 individuals molted). All molted nymphs reached stage V, which occurred 13 days p.i.. In the cold treatment, only one molt was observed in the 18 ± 0 °C treatment. The GAMLSS model showed a good fit to the data (AIC 318.76; *n* = 438).

## Discussion

The major findings of this study were that temperature increases significantly decreased the EIP of *T. cruzi* within *T. infestans*, while temperature variability did not show a significant effect (Fig. 1), and that the probability of positivity of the dejections changed with temperature, but not with variability (Fig. 4). These results showed different patterns in parasite load over time between the different temperature treatments, both constant and variable (Fig. 3).

### Temperature effects on EIP

A reduction in EIP owing to high environmental temperatures has been observed in three different species of triatomines, including *T. infestans* [55–58, 75], which is also supported by our results. In a previous study, *T. cruzi* parasites reared in vitro at four different temperatures (21 °C, 24 °C, 27 °C and 30 °C) increased in number in direct relation to temperature [75]. However, in vitro experiments lack biological components present in vectors, such as immune responses and gut microbiota, which strongly influence parasite development in vivo [76, 77].

We did not observe that daily thermal variation of 10 °C affected the extrinsic incubation period of *T. cruzi* in *T. infestans*. This lack of effect likely reflects thermal plasticity and local adaptation in both the vector and parasite. Populations exposed to heterogeneous thermal environments are expected to evolve physiological strategies that allow them to survive across broad temperature ranges [52, 78]. This is consistent with *T. infestans* and *T. cruzi* inhabiting regions such as Chaco in Argentina [79, 80], where temperature fluctuations widely exceed those applied in our temperature variable treatments. Moreover, *T. cruzi* experiences temperature shifts throughout its life-cycle due to the ectothermic nature of its vectors and their periodic ingestion of warm blood meals [81, 82], which may favor parasite tolerance to thermal variability [83].

The EIP was also affected by the amount of blood ingested, with a negative relationship between the amount of infected blood ingested and the time of the first infectious excreta (Fig. 2). Kissing bugs (subfamily Triatominae) typically consume a large amount of blood in a single meal, resulting in a relatively high intake of hemoglobin, a metalloprotein that enhances parasite replication and survival, which could explain the negative relationship between ingested blood and EIP [79]. Previous studies found that blood meal size was positively correlated with parasite concentration in the excreted urine of *Rhodnius prolixus* [54]. However, this effect diminishes with higher blood volumes, suggesting a threshold beyond which additional nutrients do not further accelerate parasite development.

### Effects of temperature on parasite load

Our study is the first to describe a bell-shaped pattern of parasite population dynamics during the first days of *T. infestans* infection. However, previous studies have reported a positive relationship between parasite load and time [55, 58], although these studies are not comparable due to differences in parasite detection (e.g. microscopy), strain [55], use of another vector species or tissue analyzed [58]. The decline in parasite load over time could be partially explained by nutrient depletion [66]. However, in our experiments, parasite load reduction continued even after re-feeding with uninfected blood (day 30 p.i.), suggesting additional regulatory mechanisms. Antibacterial protein secretion following blood ingestion [31], immune activation [84] and spatial limitation within the rectal epithelium [66] may jointly constrain *T. cruzi* multiplication. However, *T. cruzi* dynamics within triatomines involve a complex interplay that also includes alterations in the gut microbiota of insects [85]. Consequently, these interactions are highly intricate, necessitating further studies to elucidate these interactions.

Notably, 25% of the individuals (36/144) presented qPCR-negative dejections throughout the experiment. This heterogeneity reflects biological variability rather than methodological limitations, since the qPCR assay included an internal amplification control, ruling out PCR inhibition as a source of false-negative results and providing support that these negative individuals represent true biological variability in susceptibility rather than methodological artifacts. Differences in blood meal and thus infectious dose [54], gut microbiota [85], peritrophic membrane characteristics [66] and immune responses may all contribute to different susceptibility [31, 84].

Remarkably, a bell-shaped curve was observed for all temperature treatments, but with different levels of parasite load. The warm temperature treatments presented higher parasite loads than the cold temperature treatments during the entire study period (Fig. 3). Within the cold treatments, temperature variability increased parasite load, a pattern also reported in *Anopheles stephensi* infected with *Plasmodium chabaudi* [31]. Insect metabolism activation [86–88] triggered by the higher temperature from the cold variable treatment (i.e. 23 °C), which is close to the preferred range of this insect, probably explains the increase in parasite load [49, 50]. This metabolic activation could lead to *T. cruzi* activation because the number of parasites increases in direct relation to temperature [75]. The maximum daily temperature of the warm variable treatment (32 °C) may accelerate parasite development more than the minimum daily temperature (22 °C) delays it. Additionally, high temperatures weaken the immune system of triatomines, decreasing the activity of prophenoloxidase (proPO) and the enzymatic cascade of phenoloxidase (PO), both of which are involved in the defense mechanism against pathogens. This weakening of the immune system could influence the degree of earlier infection within the vector, and only in the warm variable treatment, since it reached 32 °C during the day; the other thermal treatments did not reach temperature values that could affect the proPO response [79, 89].

Environmental variability—with other factors—could potentially influence individual susceptibility to *T. cruzi* infection because coping with temperature fluctuations probably has a negative impact on the immune system of these insects. Previous studies have shown a decrease in the survival and fecundity of *R. prolixus* in thermally variable environments [13] and a decreased low-temperature performance of *T. infestans* [12].

### Effects of temperature on emitting positive dejectas

We found a significant effect of temperature on the probability of emitting positive dejecta, whereas temperature variability had no detectable effect. There was a greater heterogeneity in the probability of finding positive dejections in insects at low temperatures, whereas all insects tested positive at warmer temperatures shortly after infection. This result suggests that individual variation impacts the probability of emitting positive dejecta. Importantly, this study did not directly assess hindgut infection through dissection; therefore, parasites may have been replicating in the gut before they were detectable in dejections. It is possible that the interaction between temperature and parasite load influences the likelihood of obtaining positive samples, resulting in observed heterogeneity. Thus, environmental temperatures play a crucial role in mediating the outcome of host–parasite interactions, and these interactions are multifactorial and complex.

### Limitations

Regarding the limitations of this study, we performed all infections with a single *T. cruzi* strain, and strain differences in EIP, parasite load and infection probability have been shown in other triatomines [58, 90, 91]. Our study model was *T. infestans*, and other triatomine species may have different optimal ranges or strain susceptibilities [92, 93]. Additionally, we used colony IV nymphs, whereas field individuals would have higher genetic variability, and other developmental stages may exhibit different resistance to rising temperatures [46, 94–97]. Ultimately, these limitations could be addressed by evaluating parasite load in infected triatomines under natural environmental conditions to determine whether the laboratory-observed patterns translate to natural transmission dynamics.

## Conclusions

In this study, we show that environmental temperature (warm and cold), along with temperature variability, led to an increase in the parasite load on *Triatoma infestans*, modifying its EIP, which can potentially alter the basic reproductive number ®_0_) of Chagas disease and the number of secondary cases [18–20]. In the context of climate change, this vector may become more effective in transmitting *T. cruzi*. Nevertheless, it is important to acknowledge that temperature fluctuations can affect the survival and vital rates of insects, especially when these fluctuations are outside their optimal thermal thresholds [12, 13, 26, 89, 98]. Other factors may play a role in climate change, such as changes in precipitation and extreme climatic events, which may alter triatomine population parameters. Nonetheless, we hope that these results will contribute to a more complete understanding of the impact of increased temperature and its variability on this triatomine vector of *T. cruzi*.

## Supplementary Information

Additional file 1: Table S1. Description of fitted statistical models for each analyzed response variable.** Table S2.** List of GLM models fitted of *Trypanosoma* *cruzi* extrinsic incubation period in *Triatoma infestans*.** Table S3.**Tukey's post hoc test results for the extrinsic incubation period of *Trypanosoma cruzi* in *Triatoma infestans*.** Table S4. **List of GAM models fitted of *Trypanosoma cruzi *parasitc load in *Triatoma infestans*.** Table S5.** List of GAMLSS models fitted for positive dejections in *Triatoma infestans*.** Figure S1.** Results of interaction terms on parasitic load.

## Data Availability

Data supporting the main conclusions of this study are included in the manuscript.
