# Supplementary material for: Temperature variability increases Trypanosoma cruzi load but not the extrinsic incubation period in Triatoma infestans
Source: Parasit Vectors. 2026 Feb 2;19:101. doi: 10.1186/s13071-026-07270-y (PMC12954994; doi:10.1186/s13071-026-07270-y)
Supplement: Supplementary file 1 — Additional file 1: Table S1. Description of fitted statistical models for each analyzed response variable. Table S2. List of GLM models fitted of Trypanosoma cruzi extrinsic incubation period in Triatoma infestans. Table S3.Tukey's post hoc test results for the extrinsic incubation period of Trypanosoma cruzi in Triatoma infestans. Table S4. List of GAM models fitted of Trypanosoma cruzi parasitc load in Triatoma infestans. Table S5. List of GAMLSS models fitted for positive dejections in Triatoma infestans. Figure S1. Results of interaction terms on parasitic load. [file 13071_2026_7270_MOESM1_ESM.docx]

**Supplementary material**

**Table S1.** Description of fitted modesl for each analyzed response variable and their corresponding predictor variables.

| **Response variable** | **Variable type** | **Predictor variables** | **Model** |
| --- | --- | --- | --- |
| Extrinsic incubation period (EIP) | Days (continuous) | - Temperature treatment(T) - Body mass before infection(mb) - Ingested blood (bi) - Mouse Parasitemia (Pm) | GLM  (Gamma function) |
| Parasite load in *T. infestans* dejections | Parasitic Load (par-eq/ml)  (continuous) | - Temperature treatment (T) - Body mass before infection (mb; cubic spline*) - Ingested blood (bi; cubic spline) - Mouse Parasitemia (Pm) - Days (Days; cubic spline by treatment) - Random effect (ID)** | GAM |
| Probablility of positive dejections | Dejections positivity  (binary) | - Thermal treatment (T) - Body mass before infection (mb; cubic spline*) - Ingested blood (bi; cubic spline) - Mouse Parasitemia (Pm) - Days (Days; cubic spline by treatment) - Random effect (ID)** | GAMLSS  (binomial with RS algorithm) |

*For GAM and GAMLSS models, fitting a cubic spline allows the relationship between the response variable and a predictor to take a flexible, data-driven shape. When the spline is fitted by treatment (or another factor), the shape of the curve is allowed to vary among thermal treatments.

**Random effect (ID) accounting for repeated measurements from the same individual

**Table S2**. Model selection for GLM models of *Trypanosoma cruzi* EIP in *Triatoma infestans* acclimated to four temperature treatments that differed in temperature mean and temperature variability.

| **Models for EIP** | **df** | **LRT** | **AIC** |
| --- | --- | --- | --- |
| ~ T+ Pm + bi + mb + bi x mb | 9 | - | 700.33 |
| ~ T + bi + mb + bi x mb | 8 | 0.9733 | 698.34* |
| ~ T+ bi + mb | 7 | < 0.01 | 708.33 |
| ~ bi + mb + bi x mb | 5 | < 0.01 | 725.00 |
| ~ T | 5 | < 0.01 | 722.41 |

GLM: Generalized linear model; EIP: Extrinsic incubation period; df: degrees of freedom; LRT: likelihood ratio test; AIC: Akaike's information criterion; T: Temperature treatments; Pm: mouse parasitemia; bi: ingested blood; mb: body mass before infection. * selected model.

**Table S3**. Tukey test for EIP GLM model

|  | Estimate | Std. Error | z value | p |
| --- | --- | --- | --- | --- |
| 18 ± 5 ºC - 18 ± 0 ºC | 0.005 | 0.007 | 0.632 | 0.580 |
| 27 ± 0 ºC - 18 ± 0 ºC | 0.050 | 0.011 | 4.525 | < 0.001 |
| 27 ± 5 ºC - 18 ± 0 ºC | 0.037 | 0.010 | 3.819 | < 0.001 |
| 27 ± 0 ºC - 18 ± 5 ºC | 0.045 | 0.011 | 3.994 | < 0.001 |
| 27 ± 5 ºC - 18 ± 5 ºC | 0.032 | 0.010 | 3.214 | < 0.005 |
| 27 ± 5ºC - 27 ± 0 ºC | -0.013 | 0.012 | -1.058 | 0.580 |

**Table S4.** Model selection for GAM models of *T. cruzi* parasitic load in *Triatoma infestans* acclimated to four temperature treatments that differed in temperature mean and temperature variability. The one in bold is the selected model.

| GAM models for parasitic load | df | AIC |
| --- | --- | --- |
| ~ T + s(ID) + s(Days, by = T) + te(mb, bi, by = T) + Pm | 46.632 | 1452.900 |
| ~ T + s(ID+ s(Days, by = T) + te(mb, by =T) | 45.858 | 1448.647 * |
| ~ s (ID) + s(Days, by=T) + te(mb, bi, by =T) | 53.985 | 1482.309 |
| ~ T+ s (ID)+ s(Days, by=T) + te(mb, bi) | 34.610 | 1421.588 |
| ~ T + s(ID)+ s(Days) + te(mb, bi, by= T) | 34.750 | 1426.263 |
| ~ T + s(ID)+ s(Days) + te(mb, bi) | 33.889 | 1554.681 |
| ~ T + s(ID)+ s(Days) + te(mb, bi, by= T) | 38.313 | 1323.659 |
| ~ T + s(ID)+ Days + te(mb, bi, by = T | 33.889 | 1428.384 |
| ~ T + s(ID, + s(Days, by = T) + s(mb, by = T) + s(bi, by = T) + Pm | 52.028 | 1286.549 |
| ~ T + s(ID)+ s(Days, by = T) + s(mb, by = T) + s(bi, by = T) | 53.077 | 1284.569 |
| ~ s(ID,) + s(Days, by = T) + s(mb, by = T) + s(bi, by = T) | 52.847 | 1284.406 |
| ~ s(ID)+ s(Days, by = T) + s(mb) + s(bi, by = T) + Pm | 43.893 | 1296.872 |
| ~ s(ID) + s(Days, by = T) + s(mb) + s(bi, by = T) | 44.861 | 1290.069 |
| ~ s(ID)+ s(Days, by = T) + mb + s(bi, by = T) | 41.999 | 1296.056 |
| ~ s(ID ") + s(Days, by = T) + mb + s(bi) | 24.126 | 1330.877 |
| ~ s(ID) + s(Days) + s(mb, by = T) + s(bi) | 24.946 | 1319.625 |
| ~ s(ID) + s(Days, by = T) + s(mb) + s(bi) | 31.630 | 1327.146 |
| ~ T + s(ID) + s(Days, by = T) + s(bi, by = T) + Pm | 43.772 | 1304.689 |
| ~ T + s(ID) + s(Days, by = T) + Pm | 21.678 | 1357.071 |
| ~ T + s(ID)+ s(Days) + s(mb) + s(bi) + Pm | 20.094 | 1330.822 |

GAM: Generalized additive model; df: degrees of freedom, LRT: likelihood ratio test, AIC: Akaike's information criterion; T: Temperature treatment; s: spline; ID: triatomine individuals; Days: time measured as days; mb: body mass before infection; bi:  ingested blood; Pm:  mouse parasitemia.

**Table S5.** Model selection for GAMLSS models for the probability of *Trypanosoma cruzi* positive dejections in *Triatoma infestans* acclimated to four temperature treatments that differed in temperature mean and temperature variability. All modes have a random effect by individual (ID).

| GAMLSS models for positive dejections | gl | AUC | AIC |
| --- | --- | --- | --- |
| ~T * cs(Days)+random(factor(ID)) * Pm + bi *mb | 78.978 | 0.988 | 325.855 |
| ~T * cs(Days)+random(factor(ID)) * Pm + bi + mb | 99.06 | 0.988 | 323.809 |
| ~T * cs(Days)+random(factor(ID)) + Pm + bi + mb | 99.06 | 0.988 | 323.809 |
| ~T * cs(Days)+random(factor(ID)) + bi * mb | 99.128 | 0.988 | 323.933 |
| ~T * cs(Days)+random(factor(ID)) + Pm + bi | 98.123 | 0.988 | 321.898 |
| ~T * cs(Days)+random(factor(ID)) + bi | 97.182 | 0.988 | 320.153 |
| ~T * cs(Days)+random(factor(ID)) | 96.498 | 0.988 | 318.761* |
| ~T + cs(Days)+random(factor(ID)) | 78.978 | 0.978 | 349.802 |
| ~T + random(factor(ID)) | 6.255 | 0.691 | 528.287 |

GAMLSS: Generalized additive model for location, scale and shape; df: degrees of freedom, AUC: Area under the ROC Curve, AIC: Akaike's criterion; T: Temperature treatment; cs: cubic spline; Days: time measured as days; ID: triatomine individuals; Pm: mouse parasitemia; bi: ingested blood; mb: body mass before infection.

**
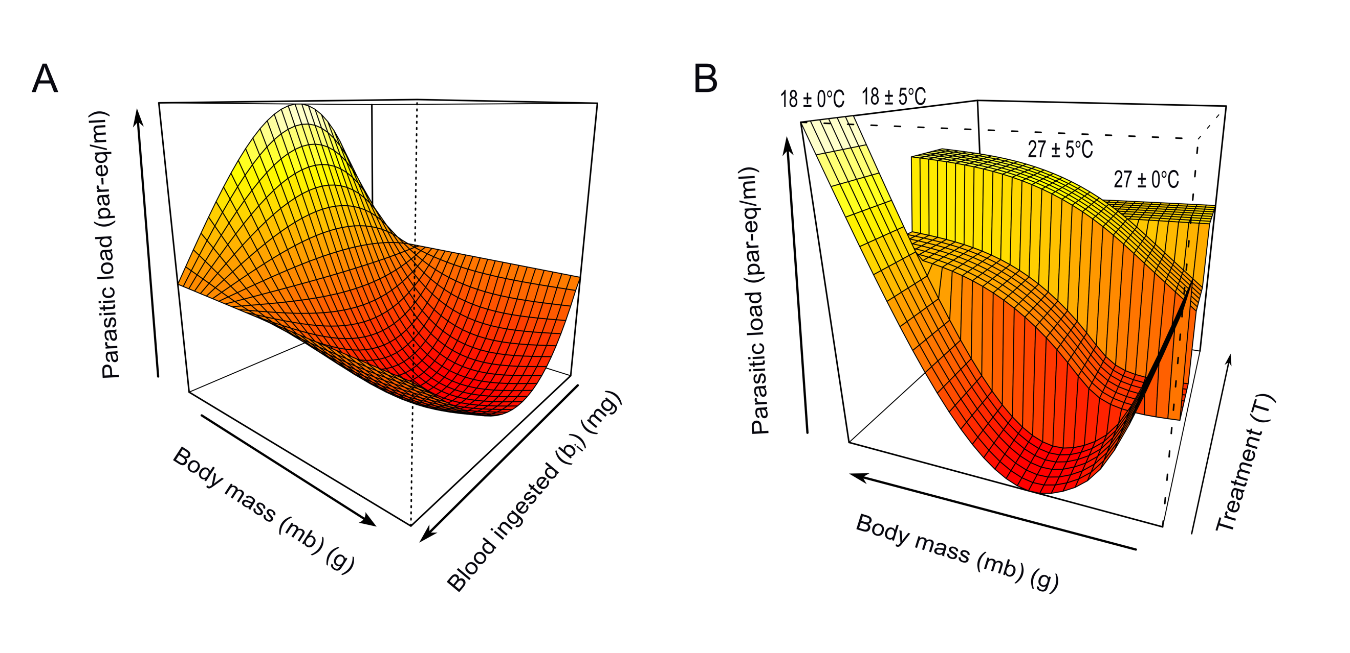
Fig. S1.** Effect of the interaction of body mass and blood ingested on parasitic load from the GAM model. mb) body mass of *T. infestans* individuals before infection in grams, bi) Blood ingested during the infection in mg.
